# A Risk-Based Approach to the COVID-19 Pandemic: The Experience in National Dental Centre Singapore

**DOI:** 10.3389/fmed.2020.562728

**Published:** 2020-11-20

**Authors:** John Rong Hao Tay, Ethan Ng, Marianne Meng Ann Ong, Chelsia Sim, Ken Tan, Chaminda Jayampath Seneviratne

**Affiliations:** ^1^Department of Restorative Dentistry, National Dental Centre Singapore, Singapore, Singapore; ^2^Oral Health Academic Clinical Programme, Duke-NUS Medical School, Singapore, Singapore; ^3^Department of Oral and Maxillofacial Surgery, National Dental Centre Singapore, Singapore, Singapore; ^4^National Dental Centre Singapore, National Dental Research Institute Singapore, Singapore, Singapore

**Keywords:** communicable diseases, emerging/epidemiology/therapy/virology, disease outbreaks, dental care/standards, infection control, dental, coronavirus infections/epidemiology/prevention & control/transmission, COVID-19

## Abstract

The emergence of a highly infectious coronavirus strain, severe acute respiratory syndrome coronavirus 2 (SARS-CoV-2), has led to a major global public health emergency. The increasing number of infected cases and fatalities worldwide forced several countries into lockdown in a bid to control virus transmission. The practice of dentistry is considered high-risk due to the generation of aerosols associated with most dental procedures, and healthcare professionals must take appropriate precautions whilst working in this challenging environment. This review aims to provide an overview on transmission routes and shares a risk-based approach to coronavirus disease 2019 (COVID-19) in a specialty tertiary center. Risk assessment and mitigation focussed on staff and patient safety, adopting a wide safety margin, and responding dynamically to the level of risk at the workplace. As the severity of the pandemic depends on many still-unknown factors and shows little sign of abating, the routine practice of dentistry will continue to be disrupted in the near future. We describe a color-coded framework to maximize safety and to minimize disease spread. Areas covered include healthcare team management, personal protective equipment, clinical work, and dental education. Guidelines in each category change with the corresponding severity of the situation, and we believe it will be useful for the safer practice of dentistry in this current climate and can be modified for future similar disease outbreaks.

## Introduction

Coronaviruses are single-stranded RNA viruses with an envelope. They were first identified around 70 years ago, and are capable of causing respiratory, gastrointestinal, and central nervous system diseases in humans and animals (1). The name “corona” implies a crown-like structure with spikes on the surface. These spike proteins are critical for binding of host cells receptors to facilitate entry, they may undergo evolutionary changes over time (2). Notable coronavirus infections in human history include severe acute respiratory syndrome-related coronavirus (SARS-CoV) in 2003 and Middle East Respiratory syndrome-related coronavirus (MERS-CoV) in 2012. SARS-CoV and MERS-CoV were highly virulent and deadly, causing ~10 and 35% mortality in infected individuals (3, 4).

The first confirmed cluster of patients with pneumonia associated with a novel strain of coronavirus virus emerged in December 2019 (5). Since then, next-generation sequencing of the virus genome has found it to be around 79% similar to SARS-CoV and 50% similar to MERS-CoV, although sufficiently divergent to be considered a novel virus (6). The official name of the disease has been termed “COVID-19,” with SARS-CoV-2 announced as the virus causing this disease (7). The announcement of COVID-19 as a pandemic by the World Health Organization (WHO) in March 2020 marked a turning point in the containment of this disease.

## COVID-19 Is A Global and National Public Health Emergency

COVID-19 was declared to be a public health emergency of international concern on 30 January 2020 (8). On 6 October 2020, WHO reported over 35 million cases globally, with over one million deaths and rising each day (9).

At the time of writing (6 October 2020) Singapore had 57,792 confirmed cases and 27 fatalities. A large majority of the confirmed cases were clinically well and were isolated and cared for at community facilities. There were 153 cases are at community facilities, 42 cases are hospitalized but are medically stable, none of the cases are in critical condition, and 57,597 cases have been discharged after successful recovery (10). A total of 88 healthcare workers and support staff were infected with COVID-19 between January and April 2020, representing 1.7% of total confirmed COVID-19 cases during the same period (11). Most of these infected healthcare workers and support staff were in the public sector (68.2%) or served at the frontlines with frequent patient contact (63.6%) (11). While most of these healthcare workers acquired COVID-19 infection locally, epidemiological links of cross-infection between healthcare workers and COVID-19-positive patients have not been demonstrated (11, 12). Singapore's management of this crisis has been based on a structured nation-wide strategy for global infectious disease outbreaks termed Disease Outbreak Response System Condition (DORSCON). This is a four-tiered color-coded crisis management plan and ranges from green, yellow, orange and red (13). The color code is based on the nature of the disease (severity and degree of spread locally and abroad) and amount of disruption to daily life on a community and individual level. The current status in Singapore since 7 February 2020 has been DORSCON orange. Accordingly, various measures such as daily health checks at workplaces, temperature screening at hospitals, and suspension of inter-school and external activities for schools were put in effect. As part of its early containment strategy, Singapore was already conducting contact tracing for confirmed cases, quarantining close contacts, issuing stay-home notices for citizens recently arriving from China, and issuing travel bans into the country to reduce the risk of imported cases into the community (14). Stay home notices were later broadened to anyone who had traveled outside Singapore, with travel bans imposed for all short-term visitors from any country (15, 16). As the transmission of COVID-19 progressed, anyone coming in from abroad were given stay-home notices or issued with quarantine orders, depending on their exposure to the virus. Others were instructed to take a leave of absence from their employers. If they presented with respiratory symptoms, they were given a mandatory 5 days of medical leave and were not allowed to leave their place of residence.

In March 2020, a suspect case was defined as one who presented with an acute respiratory illness of any degree of severity, who, within the last 14 days before onset of illness had traveled abroad to high-risk areas, or had close contact with a COVID-19-positive patient. Any person with clinical signs and symptoms suggestive of pneumonia or severe respiratory infection with breathlessness was also considered a suspect case. A close contact was defined as anyone who provided care for the COVID-19-positive patient, including a healthcare worker or a family member, or who had other similarly close physical contact; anyone who had stayed (e.g., household members) at the same place with the patient; or anyone who had close (<2 m) and prolonged contact (30 min or more) with the patient (15). Suspect cases who were medically stable were referred to a medical general practitioner for further evaluation immediately. Suspect cases who required urgent dental treatment such as the need to alleviate oral infection or pain were referred to the national specialty centers for management.

From 7 April to 1 June 2020 Singapore entered a “circuit breaker” period (Table 1) due to a surge in locally transmitted cases, new clusters developing among large groups of people housed together, and to mitigate the risk of widespread community transmission. As a result, heightened measures were implemented to minimize further community spread of the virus. Residents were prohibited from leaving their homes except for essential purposes (17). The wearing of masks was made compulsory outside one's home, and all workplaces were closed except for essential services. Elderly people and those with co-morbidities were advised to stay home as much as possible. Similarly, all elective dental procedures were halted during this period, except for essential services. Essential services were defined as procedures “if not provided or performed, would result in significant or rapid deterioration of the patient's condition, potentially threatening their health and well-being” (18). Hence, only patients requiring urgent/emergency dental care were seen.

**Table 1:** Different stages of the national response in Singapore to COVID-19.

**Disease Outbreak Response System Condition (DORSCON):** a four-tiered color-coded crisis management plan for an infectious disease outbreak and ranges from green, yellow, orange, and red. Singapore has been under DORSCON orange since February 2020 at the time of writing (6 October 2020). At NDCS, all non-essential dental review appointments were rescheduled for at least 4 months at the onset of DORSCON orange. Patients on active therapy had their treatment deferred if they presented with a specific set of respiratory symptoms, contact history, or travel history.
**Safe distancing measures:** These measures were implemented in March 2020 where seats were to be separated by at least a meter apart in public venues; places where people gathered in close contact for long periods of time such as bars, cinemas, places of worship were closed; and gatherings outside school and work were limited to 10 or fewer people. These measures were adopted at NDCS.
**“Circuit breaker” period:** A 2-month period (7 April to 1 June 2020) where heightened restrictions to community movement was imposed nationwide to further prevent community spread of SARS-CoV-2. This included the closure of non-essential workplaces and schools. Food establishments were not allowed to offer dine-in options, and all members of the public were to leave their homes only for essential needs. At NDCS, only essential services requiring urgent or emergency care were performed.
Singapore has since transited from the “circuit breaker” period to a **phased staged of reopening**. Phase 1 lasted from 2 June to 19 June 2020. Singapore is currently in Phase 2 as of 6 October 2020. Schools and business have gradually been allowed to re-open, with social gatherings of up to five people allowed. Since Phase 1 till the time of writing, NDCS requires patient appointments to be booked 15 min apart from each other. This has reduced the number of patients seen each day, reducing the potential risk of transmission between patients and staff.

The practice of dentistry is considered high-risk due to the close proximity between patients and dental practitioners and the generation of aerosols associated with most dental procedures (19). The potential for unknown interaction with COVID-19 patients due to asymptomatic spread is a concern as well. In this paper we provide an overview on transmission routes of COVID-19 and describe a risk mitigation approach during the period of DORSCON orange at National Dental Centre Singapore (NDCS), a referral tertiary center for patients needing specialist oral healthcare in Singapore. A color-coded framework to maximize safety and minimize disease spread is also described.

## Transmission Routes

The transmissibility of COVID-19 remains poorly understood. Droplet transmission from respiratory secretions is the postulated principal mechanism of spread of COVID-19. Coughing consists not only of short-range semiballistic emission, but also consists of a turbulent gas cloud and may travel as far as 7–8 m (20). Droplets may vary in size, with small particles dehydrating and existing as droplet nuclei or aerosolised forms (21). Droplet generation into the surroundings decreases significantly to background levels when a cloth is placed over the mouth during speech (22). Virus particles have been found in air exhaust outlets in a Singapore study, demonstrating airflow displacement of the virus and spread through ventilation systems (23). Airborne transmission was also shown to occur in closed spaces with air re-circulation with no evidence of contact between patients (24, 25). SARS-CoV-2 has been shown to be suspended in the air as aerosols in crowded places and may also be resuspended from medical staff personal protective equipment while they are being removed (26). The virus has also been found to be viable in aerosols for a duration of 3 h (27). The transmission distance of the virus in aerosolised form has been detected up to 4 m from COVID-19-positive patients (28). In addition, the conjunctival mucosa and bronchial epithelium appear to be portals of entry in getting infected with COVID-19 (29). This underscores the need for healthcare workers to be socially responsible and ensure that they remain appropriately masked during work, even with 1–2-m safe distancing advisories. It also implies that many routine procedures performed in dentistry are potentially hazardous due to the aerosol generation from handpieces and ultrasonic scaler tips.

Environmental contact is another possible mode of transmission. Human coronaviruses can survive on inanimate objects and can remain viable for up to 5–9 days at temperatures of 22–25°C and relative humidity of 40–50% (30, 31). Surface type seems to play a role in the viability of the virus. An experimental study using a SARS-CoV-2 strain reported viability on plastic for up to 72 h, for 48 h on stainless steel and up to 8 h on copper (27). Another study showed that the virus could still be detected on glass and banknotes after 4 days, and surgical masks, stainless steel and plastic after 7 days (32). The virus has also been found on the floor, computer mice, trash cans, and doorknobs, emphasizing the need for thorough decontamination of surfaces (28). The persistence on surfaces is particularly worrying because inadvertent self-inoculation may be possible after touching such contaminated surfaces. Some of these studies used samples of the virus that were several orders of magnitude higher than those in droplets in real-life scenarios and it may be argued that the risk of transmission of COVID-19 through surfaces is extremely small (33). However, as environmental transmission remains a theoretical possibility, disinfection of the clinical environment with prescribed workflows and trained dental personnel is prudent in reducing transmission. SARS-CoV-2019 is susceptible to standard disinfectants such as 70% alcohol, 0.5% hydrogen peroxide, 0.1% sodium hypochlorite, 7.5% povidone-iodine, 0.05% chloroxylenol, 0.05% chlorhexidine, and 0.1% benzalkonium chloride (30, 32). Decontamination can also be achieved with a combined detergent/disinfectant solution at a dilution of 1,000 parts per million available chlorine (31).

The last postulated mode of transmission is the fecal-oral route. Digestive symptoms have been found in 50% of patients with COVID-19, they may present before respiratory symptoms (34). Indeed, viable virus has been isolated from stool samples, testing positive although respiratory tract tests were negative (35). Therefore, fecal-oral transmission may occur even after viral clearance from the respiratory tract. SARS-CoV-2 has also shown to be present in toilet bowl samples in another Singapore study, strengthening the hypothesis of possible transmission through fecal matter (36).

It was initially suggested that patients with COVID-19 are not infectious until the onset of symptoms, and that the infectivity depends on the severity of their symptoms and illness (31). However, from an analysis of a group of seven clusters in Singapore it appears that transmission of SARS-CoV-2 can occur even before patients develop symptoms (37). While there are also case reports of asymptomatic transmission where patients remain asymptomatic throughout the course of infection, its exact risk of transmission is unclear, and the prevalence and detection of asymptomatic infection is not well-understood (38–40). Emerging evidence suggests that 20% of subjects that test positive for COVID-19 remain asymptomatic throughout the course of infection, and asymptomatic individuals have a relative risk of 0.35 in transmitting the disease when compared to symptomatic individuals (41). SARS-CoV-2 has an incubation period of up to 14 days, with a median time of 4–5 days before onset of symptoms (42, 43). In another study, 97.5% of persons develop symptoms within 11.5 days (44). The time for recovery after onset of symptoms generally takes 2 weeks for mild cases, and 3–6 weeks for severe cases (8, 45).

## Healthcare Team Management

In Singapore, public provision of health services (medical and dental) is through three integrated clusters, divided according to geographical location. Each cluster comprises of primary care centers, general hospitals, community hospitals and specialty centers (46). National Dental Centre Singapore (NDCS) is a tertiary dental specialist center in the Singapore Health Services cluster, and is located in the Singapore General Hospital campus, together with four other national specialty centers.

**Figure 1:**
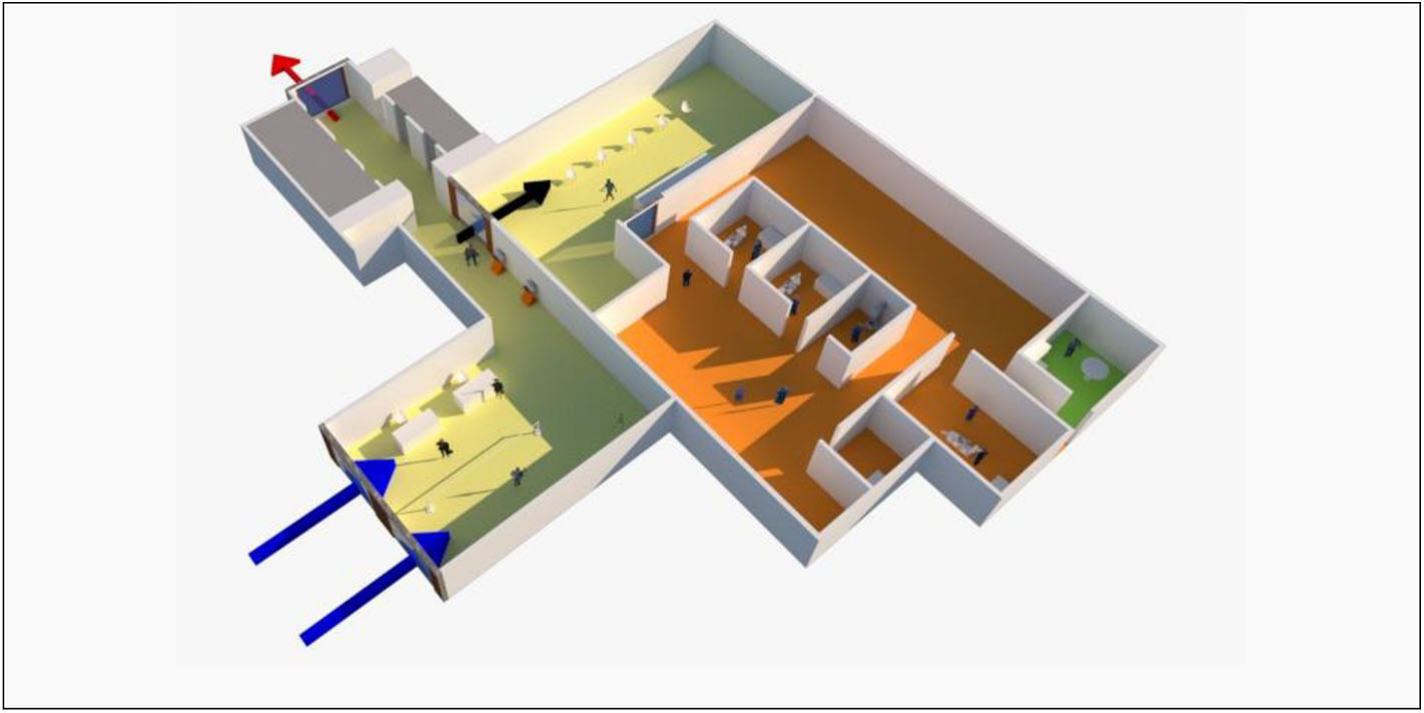
Schematic layout of the first level of National Dental Centre Singapore. Patients and staff enter through two separate centralized entrances (blue arrow on left for patients, blue arrow on right for staff). A thermal scanner is used to screen staff and patients for any signs of fever. Queues are demarcated such that patients stand at least 1 m apart. Patients are screened immediately for travel history, contact history, and for any respiratory symptoms. Known suspect cases requiring urgent or emergency care would be received and isolated at a separate holding area and treated in an isolated dental operatory unit (not shown). Patients head to the Level 1 clinic reception (black arrow) or take the lifts (demarcated gray) to clinics on the other levels. Self-check-in counters outside the clinic minimizes contact between staff and patients. Yellow floor: triage and waiting area. Orange floor: clinical area which include dental operatories (simplified as two units in this schematic), radiographic unit, decontamination room for dental instruments, and day ambulatory surgery. Green floor: pantry area for staff only. All staff and patients in the building exit through a centralized door (red arrow).

At the beginning of the COVID-19 outbreak, an important consideration was the risk of transmission unknowingly between patients and staff as there were concerns of local clusters of infection, unlinked cases, and the risk of widespread community transmission. Evidence has since shown that asymptomatic individuals that are infected with COVID-19 can have a high viral load similar to symptomatic individuals (47, 48). Both infected individuals who never develop symptoms (“asymptomatic”) or infected individuals who later develop symptoms (“pre-symptomatic”) have shown to be able to transmit the disease, with a relative risk of 0.35 and 0.63, respectively, in transmitting the disease when compared to symptomatic individuals (41). A staff member getting infected or exposed to the virus could lead to clinic downtime in its provision of core services. Movement of healthcare workers across different healthcare institutions was limited in order to mitigate this. At NDCS, dental operatories are located on the first, second, fourth, fifth and sixth levels of the building (Figure 1). The first, fifth, and sixth levels consist of closed operatories or separate rooms, while the second and fourth levels consist of both open and closed operatories. The open operatories have walls between them that are just high enough to screen patients from each other when seated upright. Day ambulatory surgeries are located on the first, third and fourth levels. Since DORSCON orange, dental specialists and general dentists were segregated to work in three independent teams, and each team had access to two levels and a day ambulatory surgery (i.e., the first team worked on first and sixth levels, the second team worked on second and third levels, and the third team worked on fourth, and fifth levels). Each team was self-contained with the complement of clinicians (specialists and general dentists), dental surgery assistants, lab technicians, patient service associate executives and health attendants. Administrative staff were similarly divided into teams during this period. Other mitigation measures included twice daily temperature taking with checks on compliance, and safe distancing of at least 1 m during all meetings and meals. Any staff member with a fever and/or acute respiratory symptoms was not allowed entry into the workplace and was advised to seek medical treatment at the staff clinic immediately. These staff members would be issued stay home notices during their medical leave period. Staff feeling unwell during the course of the day were told to stop work immediately and to seek medical attention.

To support the government's stricter measures for social and physical distancing during the “circuit breaker” period (7 April to 1 June), dental procedures were limited to emergency and urgent dental care services. The three teams in NDCS took turns to be deployed out of the center to support whole-of-government COVID-19 efforts as needed.

## Personal Protective Equipment (PPE)

A risk-based approach was adopted for the use of personal protective equipment. This depended on the risk areas in NDCS, the exposure risk, and the type of procedure performed.

At the onset of DORSCON orange, nation-wide guidelines did not advise the use of PPE for non-clinical areas with no patient contact, e.g., administrative offices and storerooms. In clinical areas (i.e., triage, reception, and operatories), the same nation-wide guidelines advised that staff members were to wear a surgical mask, and protective eyewear or a face shield for dental procedures. Treatment of suspect cases referred to national specialty centers required the use of gloves, an N95 mask, gown, and eyewear protection. A wider safety margin was adopted as a further precaution in NDCS, with all aerosol-generating procedures requiring the use of at least an N95 mask and eyewear protection, irrespective of patient status (Table 2). N95 masks had to be tested for fit prior to use as improper fits may lead to unintentional exposure of aerosols. Facial hair, in particular, may interfere with the mask seal, and in general, hair must not cross the sealing surface (31). These masks were not to be touched once put on, changed once soiled with blood or any other splash contaminants, and worn only for up to 6 h, which were similar to CDC guidelines (49).

**Table 2:** PPE use in different settings in NDCS.

**Setting**	**Personal Protective Equipment (PPE)**
Triage Staff	Surgical mask
Performing or assisting in aerosol-generating procedures	Full PPE: N95 mask, gown, gloves, and eyewear protection Powered air purifying respirators were used by healthcare workers where N95 masks were found to be ineffective during mask fitting
Performing or assisting in non-aerosol-generating procedures	Surgical mask, gown, gloves, and eyewear protection
Non-clinical areas with no direct patient contact (e.g., administrative offices, storerooms, and pantry)	Surgical masks and hand hygiene with alcohol-based hand rub if handling items from a patient environment (e.g., forms and patient files) Employees to put on surgical masks immediately after meals, conversations between staff minimized

These precautions were similar to other national guidelines regarding the use of PPE. In the United Kingdom, when caring for a patient with a suspected or confirmed case of COVID-19, dental procedures such as high-speed drilling required the use of a filtering face piece (class 3) (FFP3) respirator, a long-sleeved disposable gown, gloves, and disposable eye protection. Close patient contact of within 1 m only or in a cohorted area did not require an FFP3 respirator, but at least a fluid resistant (type IIR) surgical face mask (31). In the United States, the Centres for Disease Control and Protection recommended the use of N95 respirators or respirators that provided a higher level of protection when performing or present during aerosol-generating procedures. Furthermore, if reusable respirators such as powered air purifying respirators (PAPRs) are used, they had to be disinfected prior to reusing them (50). The use of PAPRs may provide more reliable protection, but training in putting them on and removing them without contaminating surfaces is necessary, and whether it is necessary for treating COVID-19-positive patients is unknown (51). However, others have recommended its use for other high-risk procedures such as intubation of patients (52).

Besides donning of appropriate PPE, staff members were also reminded to practice the five moments of hand hygiene, i.e., use an alcohol-based hand rub or wash hands with soap and water, (1) before touching a patient; (2) before engaging in clean/aseptic procedures; (3) after body fluid exposure risk; (4) after touching a patient; (5) after touching patient surroundings (53).

When treating suspect cases, the clinicians and dental assistant would don a full PPE consisting of an N95 mask, gown, gloves, and eyewear protection. Staff would then self-monitor for a period of 14 days for any respiratory symptoms, with twice-daily temperature monitoring. As the risk of transmission was deemed to be low due to the appropriate usage of PPE, the staff was not required to stop work.

However, staff could have unknowingly treated COVID-19-positive patients where they were later found to have the infection. If the staff members had donned appropriate PPE during treatment, the same self-monitoring guidelines were followed. However, if there was close contact without appropriate PPE, the staff would then be placed on quarantine for 14 days. If there was only casual contact (<30 min face-to-face interaction without a high risk procedure *i.e.*, consultation, making impressions, and radiographs) the staff was not required to stop work but monitored by a Ministry of Health Staff for 14 days over the phone.

## Clinical Care and Patient Management

At the onset of DORSCON orange in February 2020, in order to review PPE supply and the classification of urgent and essential dental procedures, all aerosol generating procedures were rescheduled for 3 days. A centralized patient triage and temperature screening for staff, visitors and patients was created at the ground floor of NDCS where all patients entered through one entrance before going to the waiting areas at their respective levels (Figure 1). Queues were kept fast-moving by ensuring that there were adequate staff to carry out the triage. Visitors were limited to one per patient to reduce overcrowding areas, and essential ones such as the patient's caregiver or a pediatric patient's parents took priority.

In NDCS, a wide safety margin was used in patient management. All non-essential dental review appointments were rescheduled for at least 4 months to reduce clinical workload and risk of disease transmission. Patients on active therapy that presented with any of the following were asked to have their dental procedures deferred for 2 weeks or until recovery of their symptoms if they did not present with a dental emergency: (1) any travel history in the last 14 days; (2) contact history with any known positive cases; (3) respiratory symptoms such as cough and fever. Travel and contact history were confirmed, and patients re-assessed for symptoms by the attending clinician. Suspect cases requiring urgent care were isolated at a designated holding area and managed accordingly by the dentist in an isolation room with appropriate PPE. Patients with pre-existing movement restrictions (stay-home notice, quarantine orders, leave of absence, medical leave) who presented to NDCS but had respiratory symptoms were also referred to the Department of Emergency Medicine at Singapore General Hospital for further assessment. Patients without respiratory symptoms were informed to have their treatment deferred unless they presented with a dental emergency.

On 20 March 2020, Singapore implemented stricter safe distancing measures which included separation of at least a meter between seats at public venues (54). These measures were also similarly employed at NDCS at queues and waiting areas. Patients were also encouraged to use existing self-check-in counters, in order to minimize contact with counter staff. These self-check-in counters were disinfected after every use. Alcohol-based hand rubs and posters that reminded visitors and patients to practice good respiratory and hand hygiene were also placed at these triaging and waiting room areas. This included encouraging frequent hand washing with soap and water or alcohol-based hand rubs, covering one's mouth and nose, keeping a distance of at least 1 m from someone who is coughing, sneezing, or has a fever, and to avoid touching one's eyes, nose and mouth. Precautionary measures were further heightened on 14 April 2020, and anyone leaving their homes were mandated to don a mask (55).

When Singapore entered its “circuit breaker” period, further mitigations were employed. In order to reduce the risk of non-essential commuting by patients and visitors to NDCS, only treatment requiring urgent or emergency care was continued according to specialty-determined guidelines. This included dental clearance for patients referred from other departments in the hospital receiving oncological treatment, general anesthesia, intravenous, or intramuscular antiresorptive medications. Other essential treatment included biopsies for oral pathological cases, treatment for dental trauma, periodontal abscesses, cracked teeth, and irreversible pulpitis. All non-essential cases such as orthodontic therapy, implant therapy, and asymptomatic endodontic lesions and third molars were deferred. A medication delivery system was also set up to minimize patient visits. These were patients who only required a refill of prescription for the management of chronic dental conditions, recurrent oral disorders or orofacial pain. A tele-consultation with the patient was done, and medications would be delivered to patients' homes where necessary. “Last-mile” procedures were also performed to complete ongoing treatment so as to minimize unscheduled returns arising from complications with treatment delay. For cases that required treatment, a high suction evacuator was always used during procedures that were aerosol generating, and a dental assistant was always present to ensure the practice of four-handed dentistry.

New or existing patients who had their treatment canceled or deferred also faced risks in their dental condition and overall prognoses deteriorating, such as malnutrition in frail patients awaiting denture treatment. To mitigate this, clinicians were also informed to scan through their appointment lists to identify patients who required urgent reviews and to prioritize their care concurrent to the gradual easing of restrictions of people movement and reopening of workplaces after the “circuit breaker” period.

It has to be noted that the extended suspension of routine dentistry leaves the risk of an increase in emergency or urgent cases occurring. In events where healthcare supplies (such as PPE) and manpower are strained, decision making in dentistry can be challenging. For example, it has been suggested that extractions of teeth over restorative treatment take priority in cases of acute swelling as a means of early and definitive intervention in order to reduce aerosol generation and for antibiotic stewardship (56).

## Education

Lectures, seminars, and journal clubs usually takes place within the center for dental officers, residents and staff. These are held in seminar rooms or lecture halls. When DORSCON orange started, an online refresher class on infection prevention practices was implemented for all staff. Dental education that involved different team members was conducted through video conferencing. Staff within the same teams could meet but in reduced group sizes with safety distances observed between each other. During the “circuit breaker” period, and with several staff not required to be at work, all meetings were held online.

In Singapore, large classes have been postponed in schools, including universities, due to disease transmission concerns. This has led to teaching interruptions especially for final year students. School closures have coincided with key assessment periods where internal and external assessments have been rescheduled. Medical and dental training has been disrupted in the same regard, with clinical postings for medical students suspended (57). It is crucial that continued education takes place, and amidst a time where many workplaces are closed and social distancing measures are in effect, digitized education or distance learning provides a means to mitigate the loss of learning.

Preparedness has been paramount in containing and mitigating this present pandemic, and this posture should remain so for future disease outbreaks. Singapore had a previous experience with medical and dental school disruptions during the SARS epidemic in 2003. During that period, videotaped vignettes and audiotaped webcasts for medical students were used (58). In what was novel at that time, the first fully online module was also implemented (59). Technological advances have been made since then, and with the past familiarity of the SARS epidemic, and with improved technological infrastructure, it has enabled education to continue as seamlessly as possible within the given limitations of school closures.

## Dental Practice: Quo Vadis?

There remain challenges to the practice of dentistry during this pandemic. In the absence of pharmaceutical options to treat or prevent COVID-19 cases, the only viable options for the healthcare team at large include thorough contact tracing, postponing non-urgent dental appointments for patients who are suspect cases, reducing the number of patients seen each day, and social distancing.

Implementation of these safety measures in NDCS have largely been adhered to due to strong teamwork and cooperation amongst staff members. However, the protracted nature of the COVID-19 pandemic globally has affected staff morale negatively due to restrictions in social gatherings and the inability to travel abroad to visit family members. Staff fatigue to safety measures are of concern too. Furthermore, some staff members have chosen to remain abroad to be with their families and this poses potential manpower issues in terms of work allocation. Lectures and seminars are still conducted online and its impact on the quality of dental education remains to be seen.

The severity of this pandemic depends on several still-unknown factors such as duration of acquired immunity to the virus, and seasonal variations in transmission. A gradual exit strategy from the “circuit breaker” period with long-term safe distancing measures in Singapore has been shown to be effective when compared to an immediate lifting of restrictions using an epidemic simulation model (60). However, reopening the country and its national borders for international travel risks having new community outbreaks of COVID-19. A single episode of a complete lockdown in a state or country to restrict non-essential movement and social interactions that was introduced by many countries may not be sufficient to contain the pandemic, and intermittent lockdowns of varying degrees to limit social interactions may need to continue until 2022 to prevent national critical care capacities from being exceeded, even in high-income countries (61).

With this in mind, Table 3 outlines a color-coded framework for the practice of dentistry in COVID-19. This framework offers guidelines for each specific color-code and corresponds to different suggested severities of an outbreak. This may be modified further for future similar disease outbreaks.

**Table 3:** Proposed color-coded framework for dental practice during the COVID-19 pandemic.

**Status**	**Green**	**Yellow**	**Orange**	**Red**
Nature of SARS-CoV-2	Virus has mutated into a mild form and/or does not spread easily	Virus spreads easily but: (i) has mutated into a mild form; and/or (ii) is being contained and treated effectively	Virus spreads easily and: (i) infected patients have a high morbidity/mortality rate; and/or (ii) is not being contained or treated effectively	Virus is not being contained with exponential increases of outbreaks in the community, and infected patients have a high morbidity/mortality rate
Healthcare team management	Normal deployment of staff	Normal deployment of staff	Divide staff into teams and segregate to different clinics/levels (if possible) Reduce social contact with other teams with safe distancing observed within the same team	Alternate teams on duty
Personal protective equipment	Surgical masks to be worn in operatories	Surgical masks to be worn at all times in clinical areas (i.e., triage, reception, and operatories)	N95 masks and protective eyewear for aerosol generating procedures (e.g., use of ultrasonic scalers and surgical handpieces) Surgical masks to be worn at all times in clinical areas (i.e., triage, reception, operatories, and pantry except when eating)	N95 masks and protective eyewear for aerosol generating procedures (e.g., use of ultrasonic scalers and surgical handpieces) Surgical masks to be worn at all times in clinical areas (i.e., triage, reception, operatories, and pantry except when eating)
Clinical care and patient management	Normal workload	Normal workload with heightened precautions, i.e., thorough screening at triage, monitoring temperature of patients and staff, maintain good personal hygiene Defer all suspect cases and refer to a medical GP (if medically stable) or hospital via ambulance for further management	Implement centralized patient triage Defer non-emergency cases for patients with a travel history, contact history to a known case, or respiratory symptoms Reduce patient bookings and postpone recalls Isolated holding areas and operatories for suspect cases All suspect cases and high-risk patients requiring urgent/emergency care to be treated at selected tertiary institutions or in-house hospital dental team Defer non-emergency treatment for COVID-19-positive patients	Defer all new/ongoing elective procedures Emergency cases only (e.g., swelling of the face, neck, and mouth; uncontrolled hemorrhage) Treat urgent cases (e.g., abscesses, pericoronitis, and pulpitis) with minimized aerosol generation Defer non-emergency treatment for COVID-19-positive patients Tele-consultation and medication delivery to patients' homes
Education	Normal classroom-/lecture hall-based interactions	Normal classroom-/lecture hall-based interactions	Online conferences/classes and/or classroom-based interactions with reduced group sizes, surgical masks, and social distancing	Online conferences/classes

ASA, American Society of Anesthesiologists physical status classification system; PAPR, powered air-purifying respirator.

## Conclusion

The COVID-19 pandemic is a rapidly evolving situation that is causing great disruption to daily life. Dental practice is no exception and protocols have to be adjusted to continue operation in a way that maximizes safety and reduces disease spread. SARS-CoV-2 can be transmitted through droplets, aerosols, environmental contact and oral-faecally, outlining the importance of upholding hygiene standards and minimizing contact with one another. A risk-based approach to the pandemic that is focussed on staff and patient safety, adopting a wide safety margin, and responding dynamically to the level of risks involved in the workplace is also outlined for areas in healthcare team management, personal protective equipment, clinical work, and dental education. This in turn leads to the safer practice of dentistry in the current climate.

## Author Contributions

JT and EN contributed to conception, data acquisition and drafted, and critically revised the manuscript. CJS contributed to the conception, drafted and critically revised the manuscript. MO, CS, and KT contributed to the data acquisition and drafted and critically revised the manuscript. All authors gave their final approval and agree to be accountable for all aspects of the work.

## Conflict of Interest

The authors declare that the research was conducted in the absence of any commercial or financial relationships that could be construed as a potential conflict of interest.
